# Wound healing profiles of hyperopic-small incision lenticule extraction (SMILE)

**DOI:** 10.1038/srep29802

**Published:** 2016-07-15

**Authors:** Yu-Chi Liu, Heng Pei Ang, Ericia Pei Wen Teo, Nyein Chan Lwin, Gary Hin Fai Yam, Jodhbir S. Mehta

**Affiliations:** 1 Tissue Engineering and Stem Cell Group, Singapore Eye Research Institute, Singapore; 2 Singapore National Eye Centre, 11 Third Hospital Ave, Singapore; 3 Department of Clinical Sciences, Duke-NUS Graduate Medical School, Singapore; 4 School of Material Science & Engineering and School of Mechanical and Aerospace Engineering, Nanyang Technological University, 50 Nanyang Ave, n3, 639798, Singapore.

## Abstract

Refractive surgical treatment of hyperopia still remains a challenge for refractive surgeons. A new nomogram of small incision lenticule extraction (SMILE) procedure has recently been developed for the treatment of hyperopia. In the present study, we aimed to evaluate the wound healing and inflammatory responses of this new nomogram (hyperopic-SMILE), and compared them to those of hyperopic-laser-assisted *in situ* keratomileusis (LASIK), using a rabbit model. A total of 26 rabbits were used, and slit lamp biomicroscopy, autorefractor/keratometer, intraocular pressure measurement, anterior segment optical coherence tomography, corneal topography, and *in vivo* confocal microscopy examinations were performed during the study period of 4 weeks. The corneas were then harvested and subject to immunofluorescence of markers for inflammation (CD11b), wound healing (fibronectin) and keratocyte response (HSP47). The lenticule ultrastructual changes were also analyzed by transmission electron microscopy. Out results showed that hyperopic-SMILE effectively steepened the cornea. Compared to hyperopic-LASIK, hyperopic-SMILE had less postoperative wound healing response and stromal interface reaction, especially in higher refractive correction. However, compared to myopic-SMILE, hyperopic-SMILE resulted in more central deranged collagen fibrils. These results provide more perspective into this new treatment option for hyperopia, and evidence for future laser nomogram modification.

Hyperopia, a common refractive error, has a reported prevalence of 25.2% to 31.8% in adults^1^,^2^. Compared to the treatment of myopia, which has evolved significantly since the introduction of the 193 nm excimer laser, the refractive surgical correction of hyperopia has lagged behind due to postoperative refractive regression and unpredictability^3^,^4^. Laser-assisted *in situ* keratomileusis (LASIK) is currently the most commonly performed procedure for the correction of hyperopia^5^,^6^. However, compared to myopic-LASIK, inaccuracy in the treatment and postoperative regression have been reported to be more common^3^,^5^, and the predictability and long-term stability in hyperopic-LASIK are inferior to myopic-LASIK^6^,^7^. Other options instead of LASIK include photorefractive keratectomy, and lens-based techniques such as phakic intraocular lens implantation or clear lens extraction^3^. However, no single technique has been accepted as a standard for the treatment of hyperopia^3^. The refractive surgical treatment of hyperopia therefore remains a challenge for refractive surgeons.

Refractive lenticule extraction (ReLEx) using the Visumax femtosecond system was developed in 2008 as a single femtosecond laser refractive procedure^8^. The procedure was initially introduced as femtosecond lenticule extraction (FLEx), which required the creation of a flap similar to that in LASIK with an additional posterior cut to create a refractive stromal lenticule that was then extracted^8^. In small-incision lenticule extraction (SMILE), a variation of ReLEx required no flap, the lenticule can be extracted through a small arcuate incision^9^. During the last 4 years, SMILE has become clinically available in Europe and Asia as an alternative to LASIK for the correction of myopia^10^. Due to the flapless nature of the procedure, SMILE has potential advantages over LASIK in eliminating flap-related complications, such as postoperative dry eye^11^ and flap dislocation. It also provides better corneal strength and better biomechanical stability^12^. We have previously demonstrated the wound healing advantages of ReLEx compared to LASIK, especially in the correction of high myopic refractive errors^13^.

In myopic-SMILE, the refractive lenticule is programmed to flatten the central cornea, whereas in the hyperopic-SMILE, a different nomogram is used to steepen the axial cornea. However, the nomogram adjustment to improve the laser cutting profiles for the refractive lenticule in hyperopic treatment is still under investigation^14^. Blum *et al*. reported the first results on the use of FLEx for correction of hyperopia^15^, and to our knowledge, this is the only published data on the results of hyperopic-ReLEx. The authors reported that ReLEx was a feasible and effective procedure for treatment of hyperopia, but the stability and predictability profiles were not satisfactory.

Following refractive surgery, corneal wound healing has a crucial impact on the safety, efficacy, predictability and stability of laser vision correction^16^, and hence affects the clinical outcomes. In myopic-LASIK patients, it has been reported that postoperative corneal wound healing response was associated with refractive regression^17^,^18^. Due to the fact that postoperative regression occurs even more in hyperopic correction^3^,^5^, understanding the wound healing response following hyperopic refractive surgery is important. The wound healing profiles after hyperopic-SMILE have not been previously investigated. To gain more perspective into this new refractive procedure for the correction of hyperopia, we used a rabbit model to study the effects and wound healing profiles of hyperopic-SMILE, compared to hyperopic-LASIK. The changes in corneal collagen architecture and ultrastructure after hyperopic-lenticule creation were also evaluated. We also studied the effect of surgical manipulation of lenticule extraction on the postoperative inflammatory and wound healing responses.

## Results

### Slit lamp biomicroscopy and extracted lenticule evaluation

**Figure 1:**
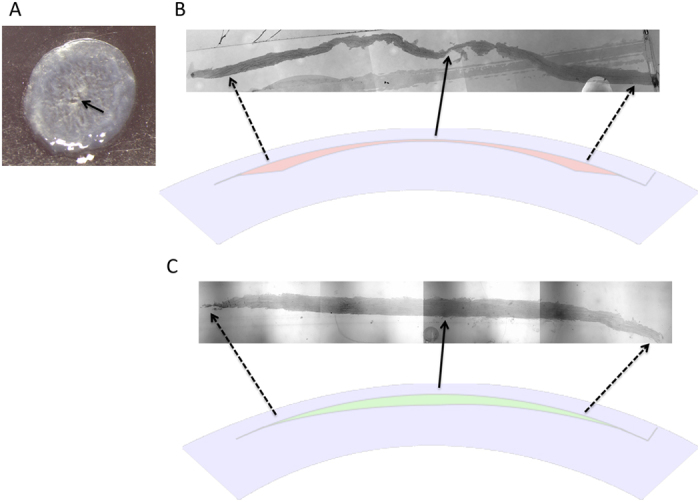
Picture of a button-hole (arrow) in the extracted hyperopic lenticule (**A**). Semi-thin tissue sections with 1% Fuchsin red staining showing the shape of an extracted +4.00 D hyperopic lenticule (**B**) and an extracted −4.00 D myopic lenticule (**C**) for comparison. The extracted hyperopic lenticule was thinnest at the central part and gradually became thicker from the center to periphery (**B**). Original magnification: 50x. The illustrations show the corneal cross section in the hyperopic-SMILE and myopic-SMILE procedures.

All procedures were uneventful, except a difficult dissection in the first two eyes in the SMILE +2.00 D group. In these 2 eyes, a buttonhole was noted in the central part of the extracted lenticule (Fig. 1A), and the data on these 2 eyes were excluded from analysis. The extracted hyperopic lenticule was thinnest at the central part and gradually became thicker from the center to periphery (Fig. 1B,C). On the slit lamp evaluation, all corneas were clear throughout the study period. There was no haze formation, flap dislocation, wound tear or other complications (Supplementary Figure 1).

### ASOCT evaluation, corneal thickness changes, and IOP evaluation

A demarcation line with increased reflectivity was seen at the flap or cap interface at all time points, but it was less apparent in the SMILE-W groups. The central thickness was comparable among different groups preoperatively (*P* = 0.672). After the surgery, the central cornea thickness remained almost unchanged in the SMILE-W2D and SMILE-W4D groups, whereas the central thickness changed by −29.4 μm, −38.7 μm, 2.4 μm and −9.8 μm in the SMILE +2.00 D, SMILE +4.00 D, LASIK +2.00 D and LASIK +4.00 D groups, respectively (Table 1).

The IOP changes after the surgery are summarized in the Supplementary Table 1. Except the control group, the mean IOP 4 weeks after the surgery was slightly but not significantly lower than that before the surgery.

### Autorefractor/keratometer and Visante Omni evaluation

**Figure 2:**
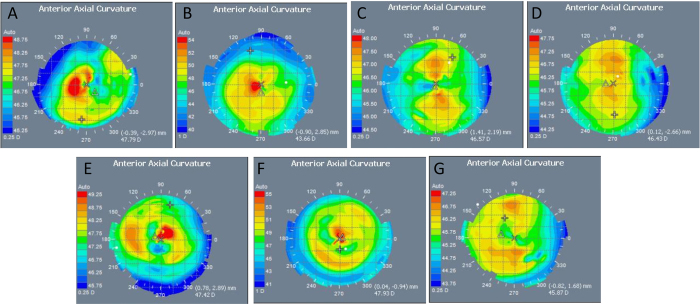
Representative anterior axial curvature maps of ATLAS corneal topographer images at 4 weeks postoperatively for the SMILE +2.00 D (**A**), SMILE +4.00 D (**B**), SMILE-W2D (**C**), SMILE-W4D (**D**), LASIK +2.00 D (**E**), LASIK +4.00 D (**F**), and control (**G**) groups. Central or paracentral steepening was observed in the SMILE +2.00 D, SMILE +4.00 D, LASIK +2.00 D, and LASIK +4.00 D eyes, with more evident changes after +4.00 D corrections (note: the maximal color scale in each image was different). The corneal curvature in the SMILE-W2D and SMILE-W4D eyes remained unchanged and was comparable to that in the control.

The mean values of keratometric measurements at different time points are shown in Table 2. At 4 weeks, the keratometric value remained almost unchanged in the SMILE-W2D and SMILE-W4D groups, whereas it changed by 2.1 D, 3.7 D, 2.4 D, and 4.6 D in the SMILE +2.00 D, SMILE +4.00 D, LASIK +2.00 D and LASIK +4.00 D groups, respectively. On the anterior axial curvature maps of ATLAS corneal topographer images, a central and paracentral steepening was observed in the SMILE +2.00 D, SMILE +4.00 D, LASIK +2.00 D, and LASIK +4.00 D eyes at 4 weeks postoperatively, with more evident changes after +4.00 D corrections. The anterior corneal curvature in the SMILE-W2D and SMILE-W4D eyes remained unchanged and was comparable to that in the controls (Fig. 2).

### *In vivo* confocal micrographs analysis

**Figure 3:**
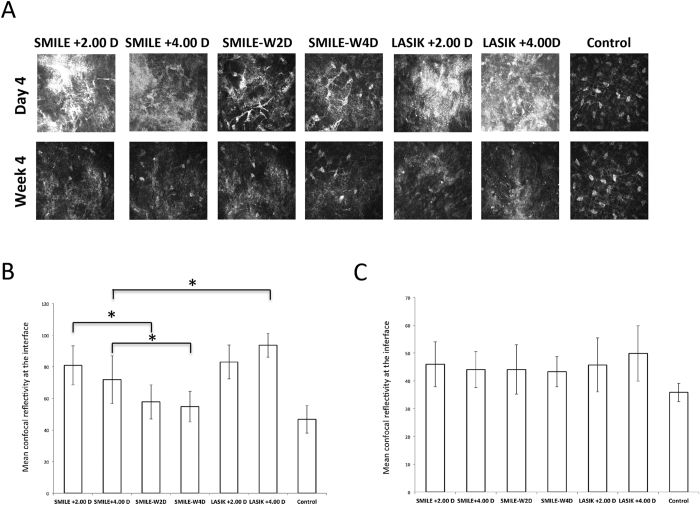
*In vivo* confocal microscopy evaluation at 4 days and 4 weeks postoperatively at the lenticule plane (SMILE and SMILE-W groups) or flap interface (LASIK group) (**A**). These planes were acellular and characterized by light-scattering particles in the post-SMILE and post-LASIK eyes at day 4. Bar graphs showing the mean intensity of stromal reflectivity for different groups at day 4 (**B**) and week 4 (**C**). At day 4, the +4.00 D hyperopic-LASIK group had significantly higher reflectivity than the +4.00 D hyperopic-SMILE group, whereas there was no significant difference between the SMILE +2.00 D and LASIK +2.00 groups. The SMILE groups had significantly higher reflectivity than the SMILE-W groups for both +2.00 D and +4.00 D corrections at day 4. These differences were not seen at week 4. Error bars represent SD; *indicates *P* < 0.05.

**Figure 4:**
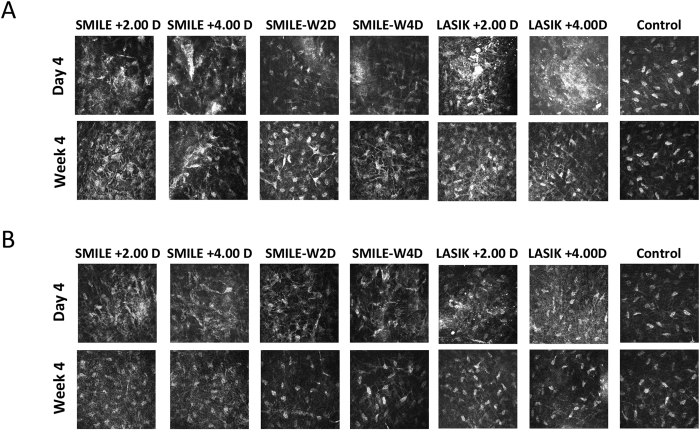
*In vivo* confocal microscopy evaluation at 4 days and 4 weeks postoperatively at the planes anterior (**A**) and posterior (**B**) to the lenticule plane (SMILE and SMILE-W groups) or flap interface (LASIK group). Activated keratocytes with high intensity of reflectivity were seen at day 4 in the post-SMILE and post-LASIK eyes for both refractive corrections. At week 4, the keratocyte nuclei became more quiescent.

Four days after surgery, the extracted lenticule plane in the SMILE +2.00 D and SMILE +4.00 D groups, or the flap interface in the LASIK +2.00 D and LASIK +4.00 D groups, were acellular and characterized by light-scattering particles (Fig. 3A). More intense and abundant reflectivity was observed in the corneas that underwent +4.00 D hyperopic-LASIK compared with those that underwent +4.00 D hyperopic-SMILE, and the difference was statistically significant after semi-quantitative analysis of the intensity of reflectivity (*P* = 0.045; Fig. 3B). When comparing the SMILE and SMILE-W groups, the post-SMILE eyes had significantly higher stromal reflectivity (*P* = 0.036 and *P* = 0.040 for the +2.00 D and +4.00 D corrections, respectively). These differences were not seen at 4 weeks (Fig. 3C). At the planes anterior and posterior to the lenticule plane or flap interface, activated keratocytes with high reflectivity were seen at day 4 in the post-SMILE and post-LASIK eyes for both refractive corrections. At week 4, the keratocyte nuclei became more quiescent (Fig. 4).

### Histological analysis

The small peripheral incision or flap incision was healed by the regrowth of new epithelium within 1 week. No apparent inflammatory cells or fibrotic scar tissue was observed in the entire cornea (Supplementary Figure 2).

### Immunohistochemistry (IHC) assays

**Figure 5:**
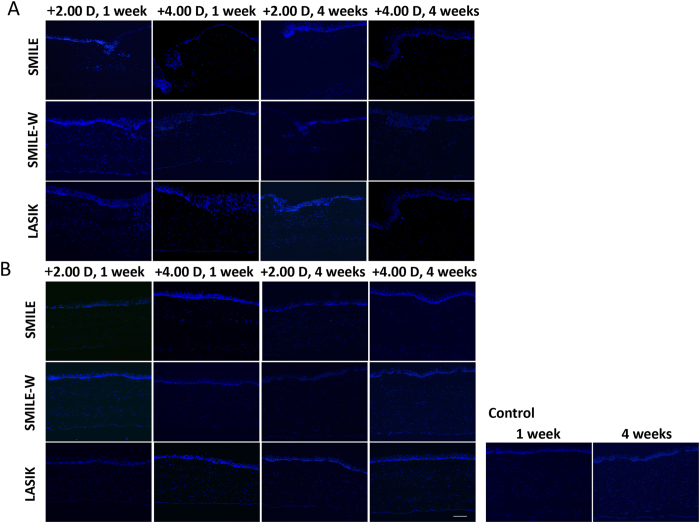
Expression of CD11b at the small peripheral incision or at the flap vertical incision (**A**), and at central corneas (**B**). CD11b-positive cells were negligible at both week 1 and week 4. Nuclei were counterstained with DAPI (blue). Original magnification: 100X, scale bar 50 μm.

**Figure 6:**
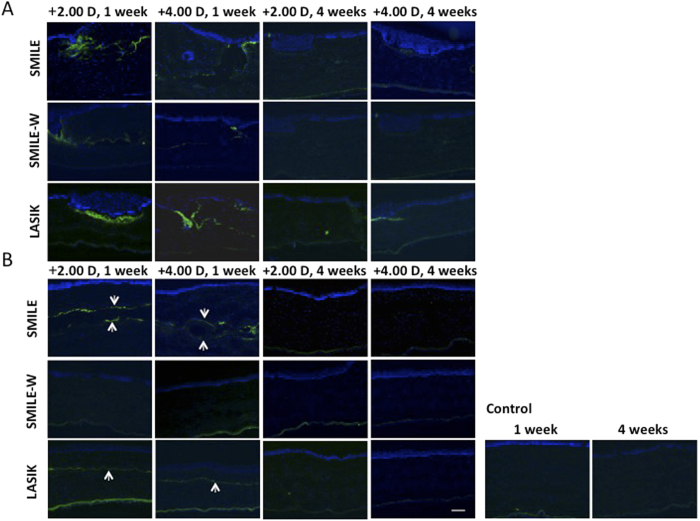
Expression of fibronectin at the small peripheral incision or at the flap vertical incision (**A**), and at central corneas (**B**). The expression of fibronectin was distinct in all experimental eyes at week 1 but was less apparent in the SMILE-W2D and SMILE-W4D eyes. At week 4, the expression of fibronectin became indistinct in all eyes except LASIK eyes, where faint fibronectin staining was still observed at the flap cut incision. Nuclei were counterstained with DAPI (blue). Original magnification: 100X, scale bar 50 μm.

Expression of CD11b, a marker for inflammatory response, was undetectable at the central cornea, as well as at the SMILE peripheral incision or LASIK flap cut at either week 1 or 4 (Fig. 5). The expression of fibronectin was distinct at the flap or small peripheral incision margin at week 1, and the staining intensity was less apparent in the SMILE-W groups (Fig. 6A). It also appeared along the anterior and posterior lamellar cuts of the lenticule in the SMILE +2.00 D and SMILE +4.00 D groups, and in the central ablated area in the LASIK +2.00 D and LASIK +4.00 D groups (Fig. 6B). At week 4, the expression of fibronectin appeared indistinct in all eyes except the post-LASIK eyes, in which faint fibronectin staining was still observed at the flap cut incision. The pattern and intensity of fibronectin staining were comparable between 2 refractive corrections for both SMILE and LASIK procedures.

**Figure 7:**
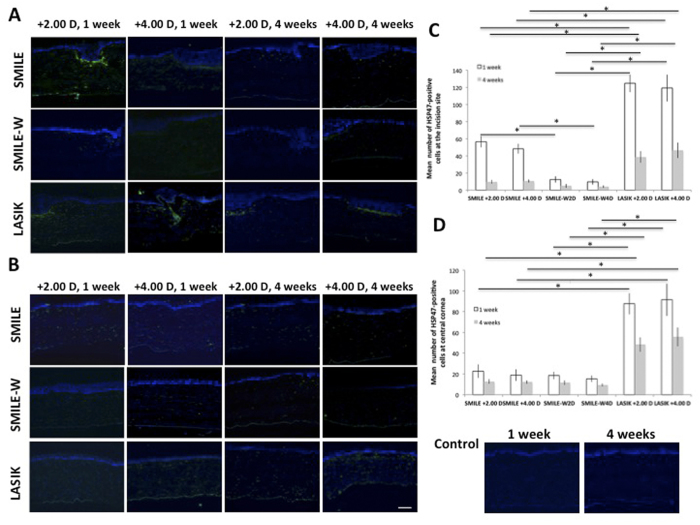
Expression of HSP47 at the small peripheral incision or at the flap vertical incision (**A**), and at central corneas (**B**). Bar graphs showing the mean number of HSP47-positive cells at the small peripheral incision or flap vertical incision (**C**), and at the central corneas (**D**). There was a significant HSP47 up-regulation at the incision site as well as at the central cornea in the hyperopic-LASIK groups (both +2.00 D and +4.00 D) than other groups throughout the study period. At the small peripheral incision site, the SMILE +2.00 D and SMILE +4.00 D groups had significantly more HSP47 expression than the SMILE-W2D and SMILE-W4D groups at week 1. Nuclei were counterstained with DAPI (blue). Original magnification: 100X, scale bar 50 μm. Error bars represent SD. *indicates *P* < 0.05.

Immunostaining of HSP47, a collagen-specific stress protein marker, showed a distinct difference between the post-LASIK and post-SMILE or post-SMILE-W eyes at both vertical incision and central cornea at week 1 and week 4. There was little to no HSP-positive cells detected at the incision site or central cornea in the SMILE-W group throughout the study period (Fig. 7A,B). Quantification of HSP47-positive cells is depicted in the bar graphs (Fig. 7C,D). When looking at the incision site, there was a significant up-regulation of HSP47 in the post-LASIK eyes than the post-SMILE and post-SMILE-W eyes (all *P* < 0.05 for both time points and for both +2.00 D and +4.00 D corrections). At week 1, the mean number of HSP-positive cells was also significantly higher in the SMILE +2.00 D and SMILE +4.00 D groups than the SMILE-W2D and SMILE-W4D groups (*P* = 0.021 and *P* = 0.033, respectively). When looking at the central cornea, significantly more positively-stained cells were present in the post-LASIK eyes than the post-SMILE and post-SMILE-W eyes (all *P* < 0.05 for both time points and for both +2.00 D and +4.00 D corrections). There was no significant disparity in the number of HSP47-postive cells between the two refractive corrections for both SMILE and LASIK procedures.

### Transmission electron microscopy evaluation

**Figure 8:**
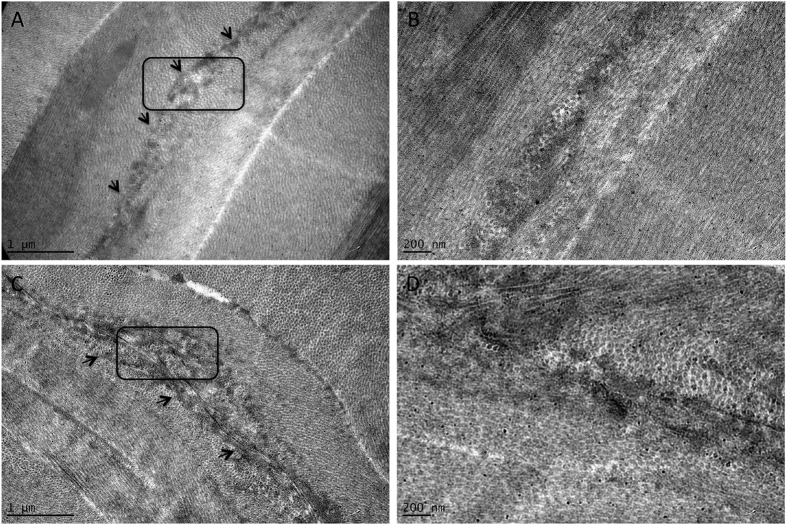
Transmission electron micrographs showing the ultrastructural changes in the central anterior surface of the intrastromal lenticule after a −4.00 D myopic-SMILE procedure (**A,B**) and a +4.00 D hyperopic-SMILE procedure (**C,D**). Figures in the right panel show the enlarged images (30000X magnification) within the black rectangles found on the left panel (10000X magnification). The anterior laser incision plane was delineated by relatively electron-dense area in both treatments (arrows; **A,C**). The border between the laser-disrupted tissue and surrounding collagen fibers was more difficult to discern, and the collagen fibers appeared more deranged in the hyperopic-SMILE group (**D**), as compared to the myopic-SMILE group (**B**).

The anterior laser incision plane was delineated by relatively electron-dense and dark area. The border between the laser-disrupted tissue and surrounding collagen fibers was more difficult to discern, and the collagen fibers appeared more deranged after the treatment with hyperopic-SMILE, compared to the treatment with the optimized myopic-SMILE nomogram (Fig. 8).

## Discussion

In the present study, we demonstrated for the first time the wound healing profiles following hyperopic-SMILE. The surgical technique for lenticule removal for hyperopic-SMILE is similar to that for myopic-SMILE, but the lenticule profiles are different. In hyperopic-SMILE, the lenticule is thinnest in the central area, and there is the presence of a transition zone, in the mid periphery outside the optical zone. In addition, the laser treatment time is longer in hyperopic-SMILE than in myopic-SMILE (6 seconds longer) due to the creation of the transition zone. These features may lead to a different postoperative inflammatory profile compared with that of myopic-SMILE. Rabbit models have been used frequently to study the wound healing response after femtosecond laser refractive surgery^18^,^19^,^20^,^21^,^22^. The corneal wound-healing rate in the rabbit is more accelerated than in the human^19^, and therefore the study time points were set at 1 and 4 weeks.

We chose +2.00 D and +4.00 D refractive corrections because it is generally recommended that hyperopic-LASIK is limited for low to moderate hyperopia, up to +4.00 D, as LASIK for high hyperopia still remains a challenge^23^,^24^. Following the initial published series, Sekundo *et al*. proposed the idea of expanding the optical zone and transition zone to reduce the risk of hyperopic regression^25^, as it would reduce the curvature gradient of the stromal surface in the treatment area and therefore reduce the postoperative corneal epithelial remodeling^25^. As compared to myopic-SMILE, hyperopic-SMILE would have a smaller clearance zone (the distance between the edge of the lenticule and the edge of the cap) due to the presence of transition zone. In their ongoing clinical study, a medium (M)-sized contact cone was used in order to maximize the total lenticule diameter^21^. However, using a larger cone (M-size) in Asian patients may increase the risk of suction loss during the lenticule creation due to the small corneal white-to-white distance and also the longer duration of lenticule creation^26^,^27^.

The post-operative corneal thickness was evaluated with RTVue ASOCT. In the present study, for the hyperopic-SMILE procedures, the minimum lenticule thickness at the lenticule center was programmed at 30 μm, and the maximum lenticule thickness was determined by the intended correction and optical zone (62 μm for the +2.00 D, and 77 μm for the +4.00 D correction). For the hyperopic-LASIK procedures, the minimum ablated thickness was set at zero at the corneal center, and the maximal ablated thickness was 46 μm for the +2.00 D, and 93 μm for the +4.00 D correction. We found that the central corneal thickness change deviated more from the programmed central lenticule thickness or programmed central ablated thickness in the +4.00 D than in the +2.00 D groups for both SMILE and LASIK procedures (the central thickness change was −29.4 μm and −38.7 μm in the SMILE +2.00 D and SMILE +4.00 D groups, and was 2.4 μm and −9.8 μm in the LASIK +2.00 D and LASIK +4.00 D groups). Whether this deviation may be associated with lower postoperative efficacy or predictability in the moderate-to-high hyperopic correction requires further clinical investigation.

Topographic central or paracentral corneal steepening was observed after hyperopic treatment, with more prominent change in the +4.00 D LASIK or SMILE procedure. The keratometric readings also showed the steeping effects. The hyperopic-LASIK eyes appeared to have a greater refractive effect, either in the +2.00 or +4.00 group, compared to the hyperopic-SMILE eyes. This maybe due to the greater thickness gradient between the center and periphery of the ablated tissue (0 to 46 μm and 93 μm for the +2.00 D and +4.00 D corrections, respectively), compared to that between the center and periphery of the lenticule (30 μm to 62 μm and 77 μm for the +2.00 D and +4.00 D corrections, respectively). It could also be due to more stromal dehydration during hyperopic-LASIK compared to hyperopic-SMILE. The difference in stromal thickness will have a secondary effect on epithelial remodeling, but a 30-μm buffer in the central corneal thickness is necessary to prevent button holing of the hyperopic lenticule following removal. In the present study, difficult dissection and the resultant buttonhole were noted in the central part of the extracted lenticule in the first two eyes, which might be related to the learning curve. The keratometric changes in the control group are within keeping of normal ageing changes in the rabbits as previously reported^28^.

*In vivo* confocal microscopy has been used to evaluate corneal stromal reaction and keratocyte activation^29^,^30^. At 4 days postoperatively, increased reflectivity was easily detected at the cap or flap interface. The intensity of stromal reflectivity was significantly higher in the post-SMILE eyes than in the post-SMILE-W eyes (both +2.00 D and +4.00 D), indicating the lenticule dissection and extraction steps induced more changes to the underlying stroma than just the laser. When comparing hyperopic-LASIK to hyperopic-SMILE groups, the LASIK +4.00 D group had significantly greater keratocyte reflectivity than the SMILE +4.00 D group, but this difference was not seen between the LASIK +2.00 D and SMILE +2.00 D groups. This may be because higher refractive correction in LASIK requires more tissue to be ablated and longer exposure to the excimer laser, hence delivering more energy to the stroma. In contrast, in SMILE procedures, the femtosecond laser simply cuts a different shaped lenticule and the energy levels do not differ significantly between different refractive corrections^13^. From day 4 to week 4, the interface stromal activity subsided in all experimental eyes.

The expression of CD11b was negligible in all experimental eyes at 1 or 4 weeks postoperatively. This result was similar to the finding in our previous study, where we demonstrated that the inflammatory response following myopic-SMILE was minimal, and the expression of CD11b only presented for the initial few days^18^. Fibronectin, produced by activated stromal fibroblasts, plays an important role in corneal wound healing process^31^. Studies showed that 1 to 2 weeks following epithelial incision, fibronectin provided a provisional matrix to support the migration of the remaining epithelial cells or fibroblasts to cover the area of defect^20^,^32^. When the wound healing response was complete, the expression of fibronectin began to decrease^20^. This can explain why fibronectin expression in our study became less distinct at week 4 in all the groups.

Heat shock protein 47 is a stress response protein^33^. It has a pro-fibrogenic role in wound healing process and serves as an inducer of collagen production in keratocytes^21^. The post-LASIK eyes had significant up-regulation of HSP47 than post-SMILE and post-SMILE-W eyes for both refractive corrections throughout the study period at both the central cornea and incision site. In comparison with SMILE, the excimer laser used in LASIK breaks molecular bonds for stromal ablation causing more tissue injury^13^,^22^. Moreover, the greater surgical manipulation from the complete flap lift in LASIK, may account for the greater extent of cell stress at the incision site. When comparing the SMILE and SMILE-W groups, the HSP47 expression was significantly higher in the SMILE eyes at the small peripheral incision during the early postoperative period, suggesting that the surgical manipulation, rather than the laser, might induce cellular stress in the surrounding stromal tissue.

In the present study, we performed TEM study to evaluate the collagen fibril arrangements after the femtosecond laser photo-disruption process. We focused on the central 2.5 mm area (i.e. within the visual axis) and the lenticule anterior surface, because the laser cut started from the lenticule posterior surface where the collagen was undisrupted. We observed that the collagen bundle arrangements and collagen fibrils around the anterior laser incision line were more deranged in the corneas treated with +4.00 D hyperopic-SMILE compared to −4.00 D myopic-SMILE. In myopic-SMILE, the anterior surface of the lenticule was cut from the center immediately after the completion of cutting the central posterior lenticule surface. In hyperopic-SMILE, there was a 6-second delay between the creation of the central posterior and anterior lenticule surfaces because of the creation of the transition zone. This delay may allow increased displacement and distortion of the anterior stromal collagen lamellae to occur as a result of tissue edema and coalescence of cavitation bubbles at the central posterior lenticule surface. The increased de-arrangements of collagen fibril, as shown in our study, may be associated with delayed early visual recovery as was seen previously during optimization of the myopic lenticule formation^34^. A nomogram modification that starts with the posterior border of the transition zone, followed by posterior border of the optical zone, followed by anterior lenticule formation, may reduce this collagen derangement. This would also allow for a reduced treatment time for transition zone from the posterior border to the anterior border.

In conclusion, we have demonstrated the wound healing response and collagen ultrastructural changes of hyperopic-SMILE, a new treatment modality for the correction of hyperopia. It effectively steepened the corneas, and the postoperative inflammation was minimal. Compared to hyperopic-LASIK, hyperopic-SMILE had less postoperative wound healing response and stromal interface reaction, especially in higher refractive correction. These may impact favorably on visual outcomes following hyperopic-SMILE, such as reducing the occurrence of refractive regression. However, compared to myopic-SMILE, hyperopic-SMILE resulted in more de-ranged central collagen fibrils, and a modification of laser firing sequence may be required to minimize this.

## Methods

### Study animals and experimental groups

Twenty-two 12- to 15-week-old New Zealand White rabbits (n = 44 eyes) were randomly allocated to four groups: hyperopic-SMILE (+2.00 D (diopters), n = 8 eyes; +4.00 D, n = 6 eyes), hyperopic-SMILE without lenticule extraction (+2.00 D, n = 6 eyes; +4.00 D, n = 6 eyes), hyperopic-LASIK (+2.00 D, n = 6 eyes; +4.00 D, n = 6 eyes), and control group (n = 6 eyes). These groups were labeled as “SMILE +2.00 D, SMILE +4.00 D, SMILE-W2D, SMILE-W4D, LASIK +2.00 D, LASIK +4.00 D, and control” groups. All animals were treated according to the guidelines of the Association for Research in Vision and Ophthalmology Statement for the Use of Animals in Ophthalmic and Vision Research. The protocol was approved by the Institutional Animal Care and Use Committee of SingHealth, Singapore. All surgeries and evaluations were performed under general anesthesia with xylazine hydrochloride (5 mg/kg intramuscularly; Troy Laboratories, Smithfield, Australia) and ketamine hydrochloride (50 mg/kg intramuscularly; Parnell Laboratories, Alexandria, Australia). All the procedures were performed by an experienced refractive surgeon (J.S.M.).

### Hyperopic-SMILE procedure

A hyperopic-SMILE correction of +2.00 D or +4.00 D was performed using a 500-kHz femtosecond laser (Visumax; Carl Zeiss Meditec, Germany). The eye was docked on a small curved interface suction cone. The intrastromal lenticule was created by first cutting the posterior surface of the lenticule using a spiral in pattern, followed by the transition zone using a spiral out pattern, and then the anterior surface of the lenticule using a spiral out pattern. The laser parameters were: 120 μm cap thickness, 7.9 mm cap diameter, optical zone 5.5 mm, transition zone 2.0 mm, central minimal lenticule thickness 30 μm, with the laser energy at 170 nJ. The spot distance and tracking spacing were set at 3.0 μm for the cap and lenticule, and at 2.0 μm for the side cuts. Side cut angles were set at 90°, incision position at 120°, and incision width was 2.5 mm. After completion of the laser firing, the cornea incision was opened with a Sinskey hook. Identification of the anterior and posterior surface edge of the lenticule was made, and the anterior surface of the lenticule was bluntly dissected with a Chansue dissector, followed by the posterior surface. The lenticule was then grasped and removed by a Tan DSAEK forceps (ASICO, Westmont, IL, USA). For the SMILE-W group, the cornea incision wound was not opened, the anterior and posterior surface of the lenticule was not dissected, and the lenticule was not removed. After the procedure, all the eyes received topical tobramycin ointment (Alcon, Fort Worth, TX, USA) 3 times daily for 1 day.

### Femtosecond laser-assisted hyperopic-LASIK procedure

LASIK flaps were created by using a 500 kHz Visumax femtosecond laser. The laser parameters were as follows: 120 μm flap thickness, 7.9 mm flap diameter, 170 nJ power, spot distance and tracking spacing of 4.8 μm for lamellar flap and 2 μm for flap side cut, flap side cut at 90°, hinge position at 90°, hinge angle of 50° and spiral in (centripetal) scanning pattern direction. After the flap was lifted, the underlying stroma underwent a hyperopic ablation of +2.00 D or +4.00 D with a 10.0 × 10.0 mm and 10.1 × 10.1 mm-treated zone, respectively, and with a 6.5 mm-optical zone using an excimer laser (Technolas; Bausch & Lomb, Rochester, NY). The excimer laser parameters were: spot size of 2.0 μm diameter, fluence of 120 mJ/cm^2^, and repetition rate of 100 Hz. When the flap was repositioned, a bandage contact lens (Bausch & Lomb) was applied and the eyelid was closed with a temporary tarsorraphy using a 6–0 silk suture. After the procedure, all the eyes received topical tobramycin ointment (Alcon, Fort Worth, TX, USA) 3 times daily for 1 day.

### Clinical evaluation

All the eyes underwent clinical evaluation with slit lamp biomicroscopy (Nikon FS-3V; Nikon), intraocular pressure (IOP) measurement using a tonopen (Tono-Pen AVIA, Reichert, NY, USA) autorefractor/keratometer (Nidek ARK-30, Hiroishi, Japan), anterior segment optical coherence tomography (ASOCT; RTVue; Optovue, Inc, Fremont, CA), Visante Omni (Carl Zeiss Meditec, Jena, Germany), and *in vivo* confocal microscopy (IVCM; HRT3; Heidelberg Engineering GmbH, Heidelberg, Germany) preoperatively, and at day 4, week 1, and weekly thereafter until week 4 postoperatively. For autorefraction, keratometry and IOP measurements, we took five consistent measurements and obtained an average. For ASOCT evaluation, three high-resolution corneal cross-sectional scans (8 mm scan length, single scan mode) were obtained for each eye at each time point. The central corneal thickness was measured by an independent observer (H.P.A). For IVCM evaluation, the central aspect of the corneas was examined with a minimum of three z-axis scans, consisting of the entire corneal thickness. For each eye, three micrographs of the lenticule plane (SMILE groups) or flap interface (LASIK group) were selected and analyzed by semi-quantifying the mean gray value of reflectivity using Image J (http://imagej.nih.gov/ij/; provided in the public domain by the National Institutes of Health, Bethesda, MD, USA)^18^,^35^. The rabbits also underwent Visante Omni scans, performed as described previously^36^.

### Histology and immunohistochemistry (IHC)

At 1 week and 4 weeks postoperatively, rabbits were euthanized under anesthesia, and the corneas were excised. The corneas were embedded in an optimal cutting temperature (OCT) compound at −80 **°**C and cryosectioned at 8 μm thickness sections. The sections were then processed for hematoxylin and eosin histochemistry and visualized under light microscopy (Axioplan 2, Carl Zeiss, Oberkochen, Germany). The immunohistochemistry staining was performed as described previously^13^. The primary antibodies were mouse monoclonal antibody against cellular fibronectin (2 μg/ml, Millipore, Billerica, MA), mouse monoclonal antibody against CD11b (4 μg/ml, BD Pharmingen, Franklin Lakes, NJ), and mouse monoclonal antibody against heat shock protein 47 (HSP47) (1 μg/ml Enzo Life Sciences, Switzerland), respectively, at 4 °C overnight. The secondary antibody was goat anti-mouse Alexa Fluor 488-conjugated (Invitrogen). After washes, sections were mounted with UltraCruz Mounting Medium with DAPI and viewed under fluorescence microscopy (Axioplan 2). Quantification of positively labeled cells was performed on 5 randomly selected non-overlapping sections at 100X magnification for each sample by a single masked observer (Y.C.L).

Moreover, for the extracted lenticule, resin-embedded semi-thin sectioning was performed to study the shape of lenticules. Another 1 rabbit with −4.00 D myopic-SMILE performed on both eyes was used for comparison. The myopic SMILE procedure was performed as we described previously^37^. These extracted lenticules were fixed with 3% glutaldehyde, washed and post-fixed in 1% aqueous solution of osmium tetroxide, and embedded in Epon-Aradite. Semi-thin sections (400 nm thick) were contrast stained with 1% Fuchsin red (Sigma) and imaged with light microscopy.

### Transmission electron microscopy

In order to study the effect of the femtosecond laser firing sequence on the lenticule creation, another three rabbits were used. A +4.00 D hyperopic-SMILE was performed on the right eyes, whereas a −4.00 D myopic-SMILE was performed on the left eyes for comparison. The intrastromal lenticules were not extracted. One day after the procedure, the rabbits were euthanized. A 2.5 mm in diameter area was excised from the central cornea and fixed in 3% glutaraldehyde (Electron Microscopy Sciences, Hatfield, PA) at 4 °C overnight. The tissue was then processed and viewed as described previously^36^.

### Statistical analysis

All data were expressed as mean ± standard deviation (SD). Statistical comparisons among different groups were performed using Kruskal–Wallis test with Dunn post-hoc tests. Statistical analyses were performed using STATA software (version 13, STATACrop, College Station, TX). *P* values less than 0.05 were considered statistically significant.

## Additional Information

**How to cite this article**: Liu, Y.-C. *et al*. Wound healing profiles of hyperopic-small incision lenticule extraction (SMILE). *Sci. Rep.* **6**, 29802; doi: 10.1038/srep29802 (2016).

## Supplementary Material

Supplementary Information

## Figures and Tables

**Table 1:** The central corneal thickness preoperatively and at 4 weeks postoperatively for different experimental groups.

	Preoperatively, μm (Pre)	4 weeks postoperatively, μm (Post)	Post-Pre, μm
SMILE +2.00 D	364.7 ± 15.9	335.3 ± 23.1	−29.4 ± 3.6
SMILE +4.00 D	365.3 ± 6.9	326.6 ± 27.2	−38.7 ± 4.4
SMILE-W2D	337.0 ± 7.5	337.1 ± 24.8	0.1 ± 1.9
SMILE –W4D	337.7 ± 14.3	342.8 ± 5.8	5.1 ± 2.4
LASIK +2.00 D	347.1 ± 32.9	349.5 ± 5.0	2.4 ± 1.4
LASIK +4.00 D	354.5 ± 4.9	344.7 ± 18.6	−9.8 ± 2.9
Control	367.8 ± 11.6	374.0 ± 12.2	6.2 ± 1.2
P value*	0.672		

^*^Comparison among different groups.

**Table 2:** The mean values of keratometric measurements preoperatively and at 4 weeks postoperatively for different experimental groups.

	Preoperatively, diopters (Pre)	4 weeks postoperatively, diopters (Post)	Post-Pre, diopters
SMILE +2.00 D	45.1 ± 1.8	47.2 ± 2.6	2.1 ± 0.9
SMILE +4.00 D	44.6 ± 1.3	48.3 ± 1.9	3.7 ± 1.1
SMILE-W2D	45.8 ± 0.6	46.1 ± 2.2	0.3 ± 0.2
SMILE –W4D	46.0 ± 1.2	46.3 ± 1.9	0.3 ± 0.3
LASIK +2.00 D	45.8 ± 0.9	48.2 ± 1.7	2.4 ± 1.1
LASIK +4.00 D	45.2 ± 1.1	49.8 ± 2.9	4.6 ± 2.4
Control	45.6 ± 1.4	44.0 ± 1.5	−1.6 ± 0.8
*P* value*	0.266		

^*^Comparison among different groups.
