# Supplementary material for: Wound healing profiles of hyperopic-small incision lenticule extraction (SMILE)
Source: Sci Rep. 2016 Jul 15;6:29802. doi: 10.1038/srep29802 (PMC4945911; doi:10.1038/srep29802)
Supplement: Supplementary Information [file srep29802-s1.pdf]

Wound healing profiles of hyperopic-small incision lenticule extraction (SMILE)

Yu-Chi Liu,[1,2] Heng Pei Ang,[1] Ericia Pei Wen Teo,[1] Nyein Chan Lwin,[1] Gary Hin Fai Yam,[1] Jodhbir S. Mehta*[1-4]

[1] Tissue Engineering and Stem Cell Group, Singapore Eye Research Institute, Singapore

[2] Singapore National Eye Centre, Singapore

[3] Department of Clinical Sciences, Duke-NUS Graduate Medical School, Singapore

[4] School of Material Science & Engineering and School of Mechanical and Aerospace Engineering, Nanyang Technological University

Correspondence:

Assoc Prof Jodhbir S. Mehta

Singapore National Eye Centre,

11 Third Hospital Avenue, Singapore 168751

Tel: +65-6227-7255; Fax: +65-6227-7290

E-mail: jodmehta@gmail.com

Supplementary Table 1. The measurement of IOP preoperatively and at 4 weeks postoperatively for different experimental groups

	**Preoperatively (mmHg)**	**4 weeks postoperatively (mmHg)**	***P* value***
**SMILE +2.00 D**	7.3 ± 1.1	6.3 ± 1.3	0.124
**SMILE +4.00 D**	7.4 ± 1.6	7.0 ± 2.0	0.166
**SMILE-W2D**	7.9 ± 1.5	7.8 ± 0.2	0.356
**SMILE –W4D**	8.2 ± 0.5	8.0 ± 0.8	0.244
**LASIK +2.00 D**	8.0 ± 1.4	7.0 ± 0.8	0.101
**LASIK +4.00 D**	8.4 ± 0.4	7.4 ± 0.5	0.184
**Control**	8.2 ± 0.2	9.0 ± 0.2	0.273

*Comparison between the measurements preoperatively and at 4 weeks postoperatively. Paired-t test.

SUPPLEMENTARY FIGURE LEGENDS

Figure S1. Representative slit lamp biomicroscopy of corneas at 4 weeks after hyperopic-SMILE (+4.00 D, A) and hyperopic-LASIK (+4.00 D, B). All the corneas remained clear, and there was no corneal haze formation throughout the study period.

Figure S2. Hematoxylin-eosin (H&E) histochemistry of corneas at 1 week and 4 weeks after surgery. The small peripheral incision or flap incision (arrows) was healed by the regrowth of new epithelium within 1 week. No apparent inflammatory cells or fibrotic scar tissue was

observed in the entire cornea. Original magnification: 10X, scale bar 100 μm.

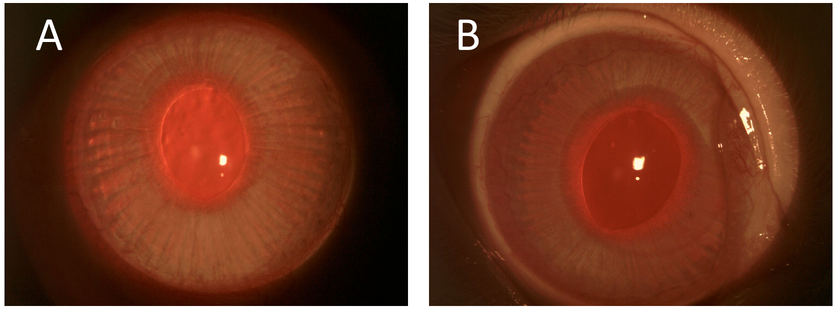
A
B

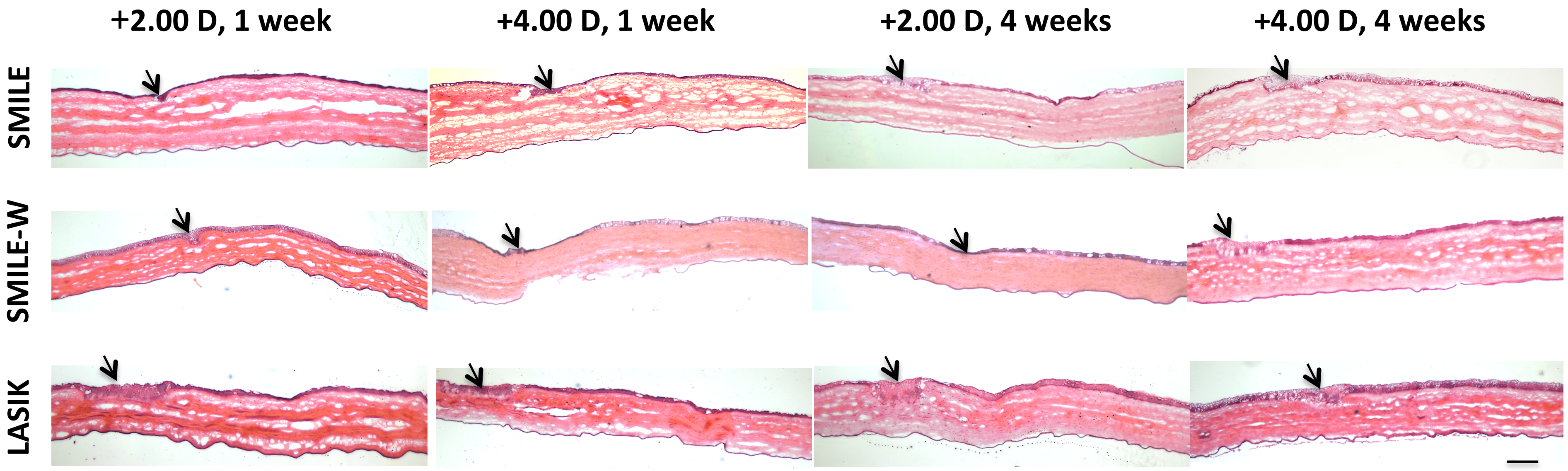

+2.00 D, 1 week
+4.00 D, 1 week
+2.00 D, 4 weeks
+4.00 D, 4 weeks
SMILE
SMILE-W
LASIK
